# Assessment of Effectiveness of 1 Dose of BNT162b2 Vaccine for SARS-CoV-2 Infection 13 to 24 Days After Immunization

**DOI:** 10.1001/jamanetworkopen.2021.15985

**Published:** 2021-06-07

**Authors:** Gabriel Chodick, Lilac Tene, Tal Patalon, Sivan Gazit, Amir Ben Tov, Dani Cohen, Khitam Muhsen

**Affiliations:** 1 Maccabi Institute for Research & Innovation, Maccabi Healthcare Services, Tel Aviv, Israel; 2 Department of Epidemiology and Preventive Medicine, School of Public Health, Sackler Faculty of Medicine, Tel Aviv University, Tel Aviv, Israel

## Abstract

**Importance:**

The BNT162b2 vaccine showed high efficacy against COVID-19 in a phase III randomized clinical trial. A vaccine effectiveness evaluation in a real-world setting is needed.

**Objective:**

To assess the short-term effectiveness of the first dose of the BNT162b2-vaccine against SARS-CoV-2 infection 13 to 24 days after immunization in a real-world setting.

**Design, Setting, and Participants:**

This comparative effectiveness study used data from a 2.6 million-member state-mandated health care system in Israel. Participants included all individuals aged 16 years and older who received 1 dose of the BNT162b2 vaccine between December 19, 2020, and January 15, 2021. Data were analyzed in March 2021.

**Exposure:**

Receipt of 1 dose of the BNT162b2 vaccine.

**Main Outcomes and Measures:**

Information was collected regarding medical history and positive SARS-CoV-2 polymerase chain reaction test and COVID-19 symptoms from 1 day after first vaccine to January 17, 2021. Daily and cumulative infection rates in days 13 to 24 were compared with days 1 to 12 after the first dose using Kaplan-Meier survival analysis and generalized linear models.

**Results:**

Data for 503 875 individuals (mean [SD] age, 59.7 [14.7] years; 263 228 [52.4%] women) were analyzed, of whom 351 897 had follow-up data for days 13 to 24. The cumulative incidence of SARS-CoV-2 infection was 2484 individuals (0.57%) during days 1 through 12 and 614 individuals (0.27%) in days 13 through 24. The weighted mean (SE) daily incidence of SARS-CoV-2 infection in days 1 through 12 was 43.41 (12.07) infections per 100 000 population and 21.08 (6.16) infections per 100 000 population in days 13 through 24, a relative risk reduction (RRR) of 51.4% (95% CI, 16.3%-71.8%). The decrease in incidence was evident from day 18 after the first dose. Similar RRRs were calculated in individuals aged 60 years or older (44.5%; 95% CI, 4.1%-67.9%), those younger than 60 years (50.2%; 95% CI, 14.1%-71.2%), women (50.0%; 95% CI, 13.5%-71.0%), and men (52.1%; 95% CI, 17.3%-72.2%). Findings were similar in subpopulations (eg, ultraorthodox Jewish: RRR, 53.5% [95% CI, 19.2%-73.2%]) and patients with various comorbidities (eg, cardiovascular diseases: RRR, 47.2% [95% CI, 7.8%-69.8%]). Vaccine effectiveness against symptomatic COVID-19 was 54.4% (95% CI, 21.4%-73.6%).

**Conclusions and Relevance:**

In this comparative effectiveness study of a single dose of the BNT162b2 vaccine, results were comparable to that of the phase III randomized clinical trial.

## Key Points

**Question:**

Is 1 dose of the BNT162b2 COVID-19 mRNA vaccine associated with protection against infection with SARS-CoV-2 and symptomatic COVID-19 in real-world settings?

**Findings:**

In this comparative effectiveness study of 503 875 individuals who received 1 dose of the BNT162b2 vaccine, the first dose of the vaccine was associated with an approximately 51% reduction in the risk of SARS-CoV-2 infections at 13 to 24 days after immunization compared with 1 to 12 days after vaccination. The first dose was associated with 54% effectiveness against symptomatic COVID-19.

**Meaning:**

The results of this study agree with vaccine efficacy as reported in the phase III randomized clinical trial after 1 dose.

## Teaser

This comparative effectiveness study examines the effectiveness associated with 1 dose of the BNT162b2 COVID-19 vaccine in a real-world setting.

## Introduction

The recently authorized BNT162b2 COVID-19 vaccine (BioNTech, Pfizer) has demonstrated 95% efficacy in preventing COVID-19 with the 2-dose regimen in a phase III placebo-controlled randomized clinical trial (RCT),^1^ with the second dose given 21 days after the first vaccine dose. The European Medicines Agency has approved the BNT162b2 vaccine, as 2 doses separated by at least 21 days, for emergency use.^2^

In light of the peaking outbreak of COVID-19, UK authorities decided to vaccinate a large number of people with high risk in the shortest possible time by postponing the second dose toward the maximal recommended vaccine dosing schedule (12 weeks).^3^ The same immunization approach is also being considered in other countries and by the World Health Organization in view of limited doses of vaccine currently available for mass vaccination.^4^ Accordingly, there is a global need to understand the real-world short-term effectiveness of the vaccine after the first dose.

The BNT162b2 RCT^1^ showed vaccine efficacy of 52% (95% CI, 29.5%- 68.4%) between the first dose and the second dose, with reduction in risk compared with the placebo starting as soon as 12 days after the first dose. This is comparable with the minimal acceptable level of efficacy of 50% in preventing COVID-19 as indicated by the World Health Organization^5^ and by the US Food and Drug Administration^6^ as one of the essential criteria to confer emergency use approval to COVID-19 candidate vaccines. However, the effectiveness of the new vaccine in protecting against infection is difficult to assess in phase III RCTs. Instead, it requires large phase IV studies in real-world settings where the vaccine is widely deployed.^5^ Furthermore, assessment of the vaccine effectiveness in real life outside of clinical trial settings is warranted, especially given the complex and unusual storage and handling requirements of BNT162b2 vaccine.

In Israel, vaccination against SARS-CoV-2 using the BNT162b2 mRNA vaccine started on December 19, 2020, with priority given to individuals aged 60 years or older, health care workers, and individuals with high risk. On January 15, 2021, Israel was ranked first in vaccination doses, with 25.3 doses per 100 capita.^7^ The aim of this study is to assess the short-term effectiveness of 1 dose of BNT162b2 in reducing infection with SARS-CoV-2 in real-world settings. In light of the RCT results, our hypothesis was that the cumulative incidence of SARS-CoV-2 infection among individuals who received the BNT162b2 vaccine would decline after 12 days after immunization compared with the incidence during the preceding 12 days.

## Methods

This comparative effectiveness study obtained ethical approval from Maccabi Healthcare Services Ethics Committee and was granted a waiver from informed consent for analysis of deidentified data. This study followed the International Society for Pharmacoeconomics and Outcomes Research (ISPOR) reporting guideline.

### Study Design and Data Sources

This comparative effectiveness study was conducted using data from a single health care system to estimate the short-term effectiveness associated with the first dose of the BNT162b2 vaccine against SARS-2-CoV-2 infection in a real-world setting. Data sources were the central databases of Maccabi Healthcare Services (MHS), a 2.6 million-member state-mandated, nonprofit, health maintenance organization (HMO) in Israel, representing one-quarter of the population of Israel. Membership in HMOs is compulsory in Israel, and by the National Health Insurance Law of 1994, all citizens must freely choose 1 of 4 national HMOs that are prohibited by law from denying membership to any Israel resident. The data set included extensive demographic data, anthropometric measurements, clinic and hospital diagnoses, medication dispensed, and comprehensive laboratory data from a single central laboratory. In Israel, everyone is assigned a unique person-specific alphanumeric identifier, called a National Health Index Number, which is used across all health systems, including in these data sets, thereby enabling data linkage.

We used data linkage to assign vaccine exposure, collect information regarding medical history and positive results in a SARS-CoV-2 polymerase chain reaction (PCR) test.^8^ PCR tests for SARS-CoV-2 are obtained from nasopharyngeal swabs. PCR testing is offered for all citizens free of charge regardless of symptoms, mostly without a need for referral, and PCR tests are widely accessible by the public (eg, through hospitals, community clinics, and drive-through and mobile walk-in testing stations).

### Study Population and End Point

Our study population consisted of all MHS members aged 16 years or older who were vaccinated during a mass immunization program from December 19, 2020, through January 15, 2021. We used as reference the results of the phase III RCT^1^ that provided experimental evidence that the BNT162b2 vaccine conferred no or little protection against SARS-CoV-2 infection until 12 days after vaccination with the first dose. Therefore, we calculated cumulative incidence of infection during a 12-day period (days 13-24 after first dose) compared with days 1 to 12 after vaccination with the first dose. Index day was thus defined as day 1 after the first dose for the day 1 to 12 period and day 13 for the day 13 to 24 period. Follow-up for infection started from the index date and lasted until the date of first SARS-CoV-2–positive PCR result, death, leaving MHS, January 17, 2021, or 12 days after index date, whichever occurred first. SARS-CoV-2 infection was defined as having at least 1 record of primary positive SARS-CoV-2 PCR test result in the MHS databases. Data regarding symptoms among individuals infected with SARS-CoV-2 were collected from electronic medical records as documented by primary care physicians at the time of referral to real-time PCR test.

Analyses excluded 2022 individuals who had a documented positive results for SARS-CoV-2 in PCR testing prior to the index date and 6389 individuals who joined MHS after February 2020 and therefore had an incomplete medical history.

### Additional Variables

Individual-level clinical and demographic data were collected from MHS central data sets. Data included age at index date, sex, and body mass index, as well as information on underlying diseases from computerized registries, including cancer, immunocompromised conditions, diabetes,^9^ cardiovascular diseases,^10^ and hypertension.^11^ The socioeconomic status (SES) index of each member’s enumeration area was based on several parameters, including household income, educational qualifications, household crowding, material conditions, and car ownership. Further information on participants’ residential areas, including characterization of ultraorthodox Jewish or Arabic communities was collected. These variables were selected based on the local epidemiological characteristics of COVID-19 in Israel showing higher incidence in ultraorthodox Jewish and Arabic communities compared with the general population and in low vs high SES communities.

### Statistical Analysis

Continuous variables were expressed as means with SDs and medians with ranges. Categorical variables were summarized as counts and percentages. Cumulative incidence plots of SARS-Cov-2 infection were created using Kaplan-Meier survival analysis and compared with the log-rank test. The comparison of the incidence of PCR-confirmed SARS-CoV-2 infection between days 1 through 12 and days 13 through 24 after immunization with 1 dose of the BNT162b2 vaccine was first estimated using a generalized linear models, applying a negative-binomial distribution with a log-link and log-time-at-risk as an offset. The offset was used to scale the counts of SARS-CoV-2 infections to daily incidence, expressed as cases per 100 000 population. The dependent variable was the number of SARS-CoV-2 cases per day during 12 days of follow-up for each group. Independent variables were sex and age category. Vaccine effectiveness (VE) was defined as infection relative risk reduction (RRR) and calculated as (1 – *relative risk*) × 100. We also calculated VE against infections with COVID-19 symptoms. Analyses were stratified by age, sex, ultraorthodox Jewish sector or community status, and comorbid condition.

The incidence of COVID-19 in Israel changed during the study period. Although Israel was under lockdown during the study period, according to Israel Ministry of Health data,^12^ there was a 33.7% increase in the number of laboratory-confirmed SARS-CoV-2 infections in adults from week 52 of 2020 to week 53 of 2020 and a 35.7% increase between week 53 of 2020 and week 1 of 2021. Since the study groups were distributed differently in calendar time, this may have attenuated our estimates of vaccine effectiveness. Therefore, sensitivity analyses included omitting first and last calendar week and censoring at the second dose of the vaccine. Individuals with a positive SARS-CoV-2 test result after the first dose are recommended to postpone their second dose. Therefore, to avoid a potential selection-bias, we did not censor the follow-up period at date of second dose. All analyses were conducted using SPSS statistical software version 27 (IBM) and R packages magrittr, readtext, dplyr, ggplot2, tidyverse, survival, and survminer (R Project for Statistical Computing). *P* values were 2-sided, and statistical significance was set at *P* < .05. Data were analyzed in March 2021.

## Results

Data of 503 875 individuals (mean [SD] age, 59.7 [14.7] years; 264 228 [52.4%] women) were analyzed, of whom 351 897 individuals had 13 to 24 days of follow-up data after first dose (Table). The study population accounts for 26% of MHS members aged 16 years or older. During the follow-up period, 49 814 individuals (9.9%) were tested for SARS-CoV-2.

**Table.** Characteristics of Study Population by Duration of Follow-up From First Dose of BNT162b2 COVID-19 Vaccine

Characteristic	Time from first dose, No. (%)	
	1-12 d (n = 503 875)	13-24 d (n = 351 897)
Age, mean (SD), y	59.7 (14.7)	61.7 (14.4)
Sex		
Men	239 647 (47.8)	170 792 (48.8)
Women	264 228 (52.4)	180 105 (51.2)
BMI, mean (SD)	27.49 (5.00)	27.38 (5.04)
SES (IQR)^a^	7 (5-8)	7 (5-8)
Residential community		
Ultraorthodox Jewish	13 936 (2.8)	9403 (2.7)
Arabic	13 084 (2.6)	7782 (2.2)
Comorbidities		
Diabetes	86 161 (17.1)	67 662 (19.2)
Cardiovascular diseases	57 057 (11.3)	46 235 (13.1)
Hypertension	186 047 (36.9)	144 296 (41.0)
Cancer	80 496 (16.0)	64 861 (18.4)
Immunosuppression	21 911 (4.3)	16 571 (4.7)

Abbreviations: BMI, body mass index (calculated as weight in kilograms divided by height in meters squared); IQR, interquartile range; SES, socioeconomic status.

^a^ Residential area SES is measured on a scale from 1 (lowest) to 10.

**Figure 1.**
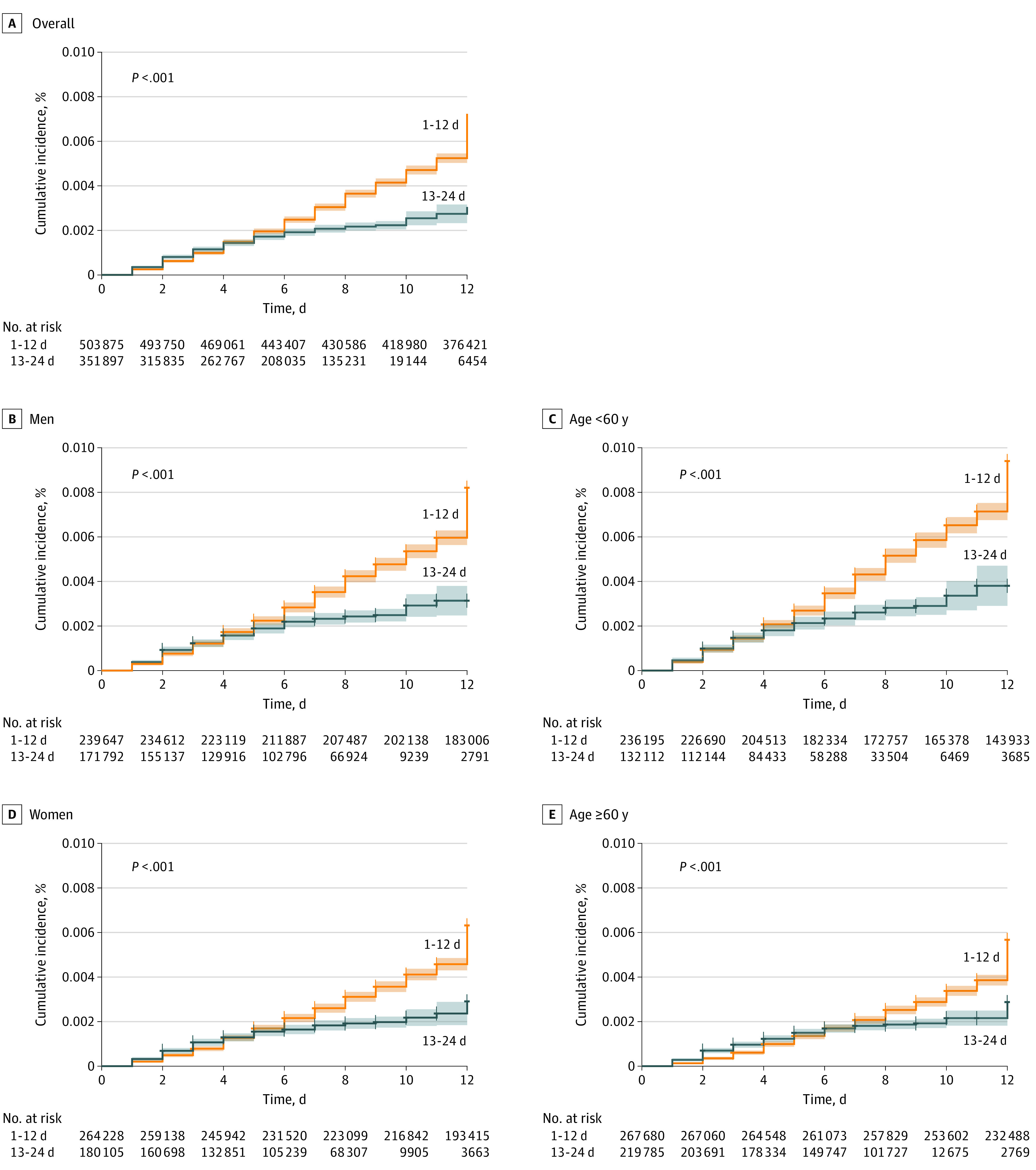
Cumulative Incidence of SARS-CoV-2 Infection by Days Since Index Date in the Period of 1 to 12 Days and 13 to 24 Days After Immunization With the First Dose of BNT162b2 Vaccine

A total of 3098 incident cases of PCR-confirmed SARS-CoV-2 infection were identified, with a cumulative risk of 0.84%, with 2484 infections (0.57%) occurring during the first follow-up period (days 1-12) and 614 infections (0.27%) occurring during days 13-24 (Figure 1). The significant decrease in incidence was evident from day 18 after first dose. Using generalized linear models, an RRR of 51.4% (95% CI, 16.3%-71.8%) was calculated with weighted mean (SE) daily incidence of SARS-CoV-2 infection declining from 43.41 (12.07) infections per 100 000 population in days 1 to 12 to 21.08 (6.16) infections per 100 000 population in days 13 to 24 after immunization.

**Figure 2.**
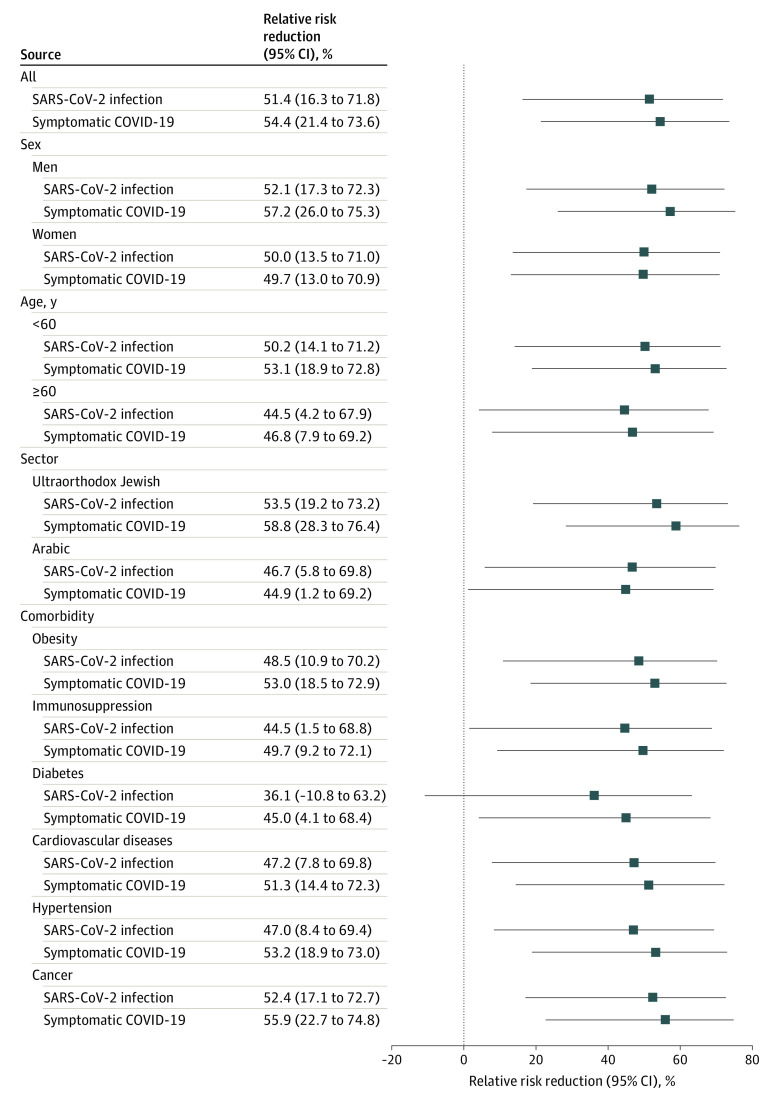
Relative Risk Reduction by Subgroup in Base Model

Similar findings were seen in stratified analyses by age group (age ≥60 years: RRR, 44.5%; 95% CI, 4.1%-67.9%; vs age <60 years: RRR, 50.2%; 95% CI, 14.1%-71.2%), sex (women: RRR, 50.0%; 95% CI, 13.5%-71.0%; vs men: RRR, 52.1%; 95% CI, 17.3%-72.2%), ultraorthodox Jewish communities (RRR, 53.5%; 95% CI, 19.1%-73.2%), and comorbidities (eg, cardiovascular diseases: RRR, 47.2% [95% CI, 7.8%-69.8%]) (Figure 2).

Limiting the analysis to infections with documented COVID-19 symptoms, a total of 1692 incident cases were observed in days 1 through 12, with a cumulative risk of 0.39% and accounting for 68% of SARS-CoV-2 infections, compared with 406 incident symptomatic COVID-19 cases during days 13 through 24, a cumulative risk of 0.17% and 66% of SARS-CoV-2 infections. The calculated VE against symptomatic cases was 54.4% (95% CI, 21.4%-73.0%). Comparable VEs were found across age and sex groups, as well as among individuals with chronic conditions (Figure 2), such as patients with diabetes (45.0%; 95% CI, 4.15%-68.4%), immunosuppression (49.7%; 95% CI, 9.2%-72.1%), and cancer (55%; 95% CI, 22.7%-74.8%).

## Discussion

In this comparative effectiveness study using real-world data, we found that the BNT162b2 mRNA vaccine was associated with 51% reduced risk of PCR-confirmed SARS-CoV-2 infection and symptomatic COVID-19 during days 13 to 24 after immunization with the first dose, compared with the preceding 1 to 12 days. While our effectiveness estimate was comparable with the 52% efficacy in preventing COVID-19 calculated in the phase III RCT of BNT162b2,^1^ there are major differences in interpreting these findings. First, unlike in the RCT, our assessment of vaccine effectiveness assessed days 13 to 24 after immunization, whereas SARS-CoV-2 infections in the phase III RCT occurred earlier. In fact, in the RCT, only 2 incident infections were diagnosed between days 14 and 21 days in the vaccine group, compared with 18 incident infections in the placebo group,^1^ indicating an efficacy of 89%

Interestingly, the BNT162b2 vaccine (at 30 µg, the same dose used in the efficacy study^1^) induced only a modest level of neutralizing antibodies in participants aged 18 to 55 years and even lower in participants aged 65 to 85 years, as measured on day 21 after the first injection of the safety and immunogenicity study.^13^ This might explain our real world estimate of 50% protection against overall SARS-CoV-2 infections for 12 to 21 days after immunization with first dose of the BNT162b2 vaccine. Analogically, the very good neutralizing antibody response after the 21-day booster as shown in the immunogenicity study^13^ is encouraging, suggesting a very good chance for high-level protection against SARS-CoV-2 infection after immunization with the second dose. It is possible that this robust systemic functional response 7 days after the second intramuscular injection of BNT162b2 (but not after a single injection) is paralleled by a significant local immune response, including both immunoglobin G of systemic origin and secretory immunoglobin A, with potential positive implications in prevention of virus transmission. Continuing assessment of effectiveness in parallel with serological and virological data in our setting could confirm or refute this assumption and support the evaluation of neutralizing antibodies as it correlates with protection against COVID-19 and SARS-CoV-2 asymptomatic infection.

Our estimation of 51% vaccine effectiveness against PCR-confirmed SARS-CoV-2 infection and 54% vaccine effectiveness against symptomatic infection 13 to 24 days after immunization with first dose of BNT162b2 provides critically needed evidence on the early performance of BNT162b2 vaccine in real life and has some important implications in decision-making to prevent transmission of SARS-CoV-2 and control the pandemic. While these results are encouraging, the BNT162b2 vaccine should be administered in a 2-dose regimen 21 days apart, as licensed for emergency use approval, to achieve maximum protection and impact in reducing the burden of COVID-19 and possibly the transmission of SARS-CoV-2.

Our study has several strengths. The automated data collection of vaccination status and laboratory results that are offered to all citizens free of charge allowed us to comprehensively explore vaccine effectives with minimal threat of the information bias that characterizes studies relying on self-reported diagnosis. By comparing only vaccinated individuals in different time intervals after immunization, we minimized potential selection and indication bias that may stem from comparing vaccinated vs unvaccinated^14^ or test-negative studies.^15^

### Limitations

This study has some limitations. As with any observational study, our study may have been affected by unreported vaccinations; however, the total number of vaccinated individuals by January 15, 2021, in our analysis accounts for approximately 25%^16^ of the total number of vaccinated individuals in Israel on that date, which is comparable with the market share of MHS, leaving little room for significant gaps in data. Despite the relatively large study population, our study was also limited by the number of individuals with chronic illness who were infected during the short study period, yielding wide 95% CIs of the calculated VE. Additional limitations are the change in health seeking behavior and decreased test rate 2 weeks after first dose, which may have caused more asymptomatic infections to go undocumented. Nevertheless, this potential information bias is likely insignificant, as VE calculated for all infections was similar or lower to that calculated for symptomatic cases.

Our follow-up period for assessing VE ended in day 24 after the first dose, 3 days after day 21, at which point the second dose can be given. This may have potentially increased the vaccine-induced immune response in the study cohort in the last days of follow-up. Nonetheless, this bias had probably only limited effect. First, according to the phase II RCT,^13^ the highest neutralization titers are reached only on day 35 after first dose. Second, our VE estimates are in line with the results of a large real-world data analysis by Dagan et al^17^ from a second HMO in Israel in which VE at days 14 through 20 after the first dose against documented infection was 46% (95% CI, 40%-51%) and against symptomatic COVID-19 was 46% (95% CI, 40%-51%) and 57% (95% CI, 50%-63%). Dagan et al^17^ compared vaccinated and unvaccinated individuals who required rigorous matching, and a significant number of unmatched individuals were excluded.

## Conclusions

In this comparative effectiveness study, a single dose of the BNT162b2 COVID-19 vaccine was associated with a 51% reduction in the incidence of SARS-CoV-2 infection during 13 to 24 days after immunization compared with the preceding 12 days. This estimate was consistent across age, sex, sector, and comorbidity. These findings have global public health implications and open new horizons toward the control of the COVID-19 pandemic and reduction of transmission of SARS-CoV-2. Global efforts should be made to accelerate COVID-19 vaccine deployment.
